# From the pandemic to the pan: the impact of COVID-19 on parental inclusion of children in cooking activities: a cross-continental survey

**DOI:** 10.1017/S1368980021001932

**Published:** 2021-05-05

**Authors:** Tony Benson, Blain Murphy, Amanda McCloat, Elaine Mooney, Moira Dean, Fiona Lavelle

**Affiliations:** 1 Institute for Global Food Security, School of Biological Sciences, Queen’s University Belfast, School of Biological Sciences, 19 Chlorine Gardens, Belfast BT9 5DL, UK; 2 School of Home Economics, St. Angela’s College, Sligo, Ireland

**Keywords:** COVID-19, Cooking, Parents, Children, Diet quality, Cross-sectional survey, Cross continental

## Abstract

**Objective:**

This study aimed to investigate the impact of COVID-19 on time spent cooking and parental inclusion of children in cooking. The secondary aim was to investigate differences between those who frequently included their children in cooking activities during the COVID-19 pandemic and those who included their children less, on a number of factors such as working from home, parents’ diet quality and cooking skills confidence.

**Design:**

Cross-continental survey with Wilcoxon-signed ranks, Independent *t* tests, Mann–Whitney *U*, *χ* ^2^ and a binomial logistic regression used for assessment.

**Setting:**

Online.

**Participants:**

A convenience sample of parents over 18 years from the island of Ireland (*n* 180), Great Britain (*n* 312), the USA (*n* 120) and New Zealand (*n* 166).

**Results:**

In three regions, parents’ time spent cooking and inclusion of children in everyday cooking activities increased (*P* < 0·001). Country (OR = 3·6, 95 % CI 1·7, 7·6), education (OR = 1·6, 95 % CI 1·1, 2·4), cooking skills confidence (OR = 1·02, 95 % CI 1·009, 1·032) and a parental higher intake of vegetables (OR = 1·3, 95 % CI 1·1, 1·5) were significant predictors of a more frequent inclusion of children in cooking activities.

**Conclusions:**

While there a number of key benefits to including children in cooking for the children such as providing life skills and increases in diet quality, this study highlighted a higher intake of vegetables by parents who included children more frequently in cooking activities. With continued lockdowns due to COVID-19 and perhaps more flexibility in working from home in the future, including children in cooking activities should be a key public health message for both children and parents.

Children’s physical and mental well-being can be severely impacted by childhood obesity^(1,2)^. Part of the rise in childhood obesity has been attributed to children’s dietary behaviours^(2)^. Consumption of home-cooked meals has been associated with a normal BMI and body fat percentage^(3)^; however, with the reported decline in home cooking, nutrition education programmes including cooking interventions are being recommended as preventative strategies^(4–6)^. Furthermore, cooking skills (CS) confidence has been associated with positive dietary patterns in adults^(7)^, and learning CS at younger ages have been associated with positive dietary outcomes^(8)^. While parents are supportive of children learning these CS and report learning their CS at younger ages^(9,10)^, time and fear have been emphasised as barriers to including children in cooking^(10)^.

The global COVID-19 pandemic has caused dramatic shifts in societal norms through movement restrictions and social distancing measures^(11)^, including moving to a ‘working from home’ (WFH) model^(12)^. Cooking and baking have been used to pass extra/leisure time in the home and to demonstrate skills on social media^(13)^. However, whether the additional time and WFH have been sufficient to overcome the barriers to including children in cooking activities is unknown. During the initial spread of COVID-19, differences in resources, law, cultures and epidemic phases meant that countries took different approaches to curtailing the spread of the virus^(14)^. These differences of approaches may have impacted time allowances and inclusion of children in the kitchen. Therefore, this study aimed to investigate the impact of COVID-19 on time spent cooking and parental inclusion of children in cooking and baking. The secondary aim was to investigate differences between those parents who included their children more frequently or less frequently in cooking activities during COVID-19 on factors such as WFH, diet quality and CS confidence.

## Methods

The participants from this study form part of a wider sample from a larger COVID-19 study on food practices^(15)^. Those participants who responded to questions relating to the inclusion of children in cooking and baking activities are included in the current study. Briefly, a cross-sectional online survey was conducted from May to June 2020, with a convenience sample of adults (inclusion criteria – >18 years) from three continents. Due to the restrictions of COVID-19, multiple recruitment strategies were used such as social media, researcher networks and panel participants from a market research agency operating in all regions (Dynata). Participants for this study, known as ‘parents’ from here, were recruited from the island of Ireland (IOI), Great Britain (GB), New Zealand (NZ) and the USA. The parent characteristics are presented in Table 1.

**Table 1:** Basic parental demographics across the different regions

Country/Region	IOI		GB		USA		New Zealand	
	*n*	%	*n*	%	*n*	%	*n*	%
Characteristic								
Total (*n*)	180		312		120		166	
Age								
Mean	40·13		43·02		39·78		40·45	
sd	9·07		12·12		16·14		12·00	
BMI								
Mean	26·74		26·52		29·06		27·25	
sd	6·46		5·59		11·31		6·27	
Isolating								
Mean	46·74		42·15		35·82		35·31	
sd	18·59		27·96		30·57		20·30	
Social distancing								
Mean	50·14		49·66		39·18		42·32	
sd	14·53		25·27		31·05		25·35	
Gender								
Male	16	8·9	153	49·0	53	44·2	79	47·6
Female	164	91·1	158	50·6	66	55·0	87	52·4
Other	0	0	1	0·4	1	0·8	0	0
Education								
None	0	0	0	0	0	0	2	1·2
Primary school	1	0·6	5	1·6	1	0·8	5	3·0
Secondary school	20	11·1	67	21·5	24	20·0	26	15·7
Additional training	41	22·8	71	22·8	17	14·2	28	16·9
Undergraduate degree	46	25·6	102	32·7	40	33·3	53	31·9
Postgraduate degree	72	40·0	67	21·5	38	31·7	52	31·3
Employment status								
Full time	98	54·4	189	60·6	68	56·7	104	62·7
Furloughed or temporarily unemployed	25	13·9	37	11·9	8	6·7	4	2·4
Part time (<8 h/week)	2	1·1	8	2·6	12	10·0	7	4·2
Part time (>8 h/week)	36	20·0	31	9·9	10	8·3	23	13·9
Retired	1	0·6	16	5·1	12	10·0	5	3·0
Long-term sickness/disability	3	1·7	3	1·0	1	0·8	3	1·8
Unemployed (either seeking or not seeking employment)	15	8·3	28	9·0	9	7·5	20	12·0
Working from home								
All working hours	79	43·9	137	43·9	55	45·8	38	22·9
Some working hours	20	11·1	36	11·5	16	13·3	36	21·7
Not working from home	37	20·6	55	17·6	19	15·8	59	35·5

### Procedure

This research is reported in line with the STROBE Statement^(16)^. SurveyMonkey was used for the administration of the survey. Parents were screened for eligibility, following information on the survey and consent. Sociodemographic information such as age, gender and education level (split into above and below university for analysis) were obtained. The survey took approximately 15 min to complete.

### Survey measures

#### Cooking-related variables

Parents were asked how often they ‘include your child in “everyday” meal preparation (such as making lunches or making dinner)’, and ‘include your child in any other cooking/baking activities’, both before and during the COVID-19 pandemic, with responses ranging from ‘Everyday’ to ‘Never’ on a six-point scale. Responses were reverse-coded so that a higher score indicated a higher frequency. Additionally, parents were asked how long they spend preparing and cooking the main meal, both midweek and at the weekend in minutes^(8)^ both before and during the pandemic. CS confidence was assessed using the validated fourteen-item measure^(17)^, where parents rated how good they were at the fourteen items on a scale of 1 to 7, with an additional option of ticking ‘Never/Rarely do it’. The items are then summed to give an overall CS confidence score. Parents were asked at what stage of their life they learnt most of their CS^(8)^ – as a child, as a teenager or as an adult. For analysis, the child and teenager responses were combined.

#### Diet quality and health indicators

Fruit and Vegetable intake were adapted from the Dietary Instrument for Nutrition Education (DINE) measure^(18)^, where parents were asked ‘About how many servings or portions per day DID/DO you eat of the following foods? (One serving could be, an apple a banana or a handful of chopped carrots or a bowl of green salad)’ and responded for ‘Vegetables (fresh/frozen/canned, excluding juice)’, and ‘Fruit (fresh/frozen/canned/dried, excluding juice)’. Parents also provided their height and weight, and BMI was calculated.

### Working from home status

Parents were also asked, ‘are you currently working from home?’; categorised ‘yes’ or ‘no’ for the analysis.

### Data analysis

Data were analysed using IBM SPSS version 25. Descriptive statistics (Mean, sd, percentages) were used for demographic data. Intra-region differences were assessed using Wilcoxon-signed ranks tests. To assess the differences between those who included their children in cooking activities more frequently than those who did less frequently during the pandemic (here in known as high includers and low includers, respectively), a median split was used. Independent *t* tests with 95 % CI and Mann–Whitney *U* tests were used to assess the differences between low and high includers for age, CS confidence, BMI, fruit intake and vegetable intake. *χ* ^2^ tests or Fishers exact test where assumptions were violated^(19)^ were used to examine relationships between low and high includers and education, gender and age when the parents learnt their skills and WFH status. A binomial logistic regression was used to examine the predictors of low/high includers. OR were adjusted for all other variables in the model. A significance level of 0·05 was used.

## Results

### Within-region differences

IOI, GB and NZ had significant increases in time spent cooking both midweek and at the weekend (*P* < 0·001), whereas no change was seen in the USA sample, Table 2. Additionally, significant increases were seen in IOI, GB and NZ in including their children in everyday cooking and baking activities (*P* < 0·001). Again, this change was not seen in the USA.

**Table 2:** Within-country differences from before the COVID-19 pandemic to during the pandemic on time spent cooking and the inclusion of children in the kitchen

	IOI (*n* 180)					GB (*n* 312)					USA (*n* 120)					New Zealand (*n* 166)				
	Before		During		Significance	Before		During		Significance	Before		During		Significance	Before		During		Significance
	Mean	sd	Mean	sd	*P*	Mean	sd	Mean	sd	*P*	Mean	sd	Mean	sd	*P*	Mean	sd	Mean	sd	*P*
Time spent cooking – midweek (min)	41·34	25·69	51·24	37·44	<0·001	32·71	29·47	39·18	34·94	<0·001	25·94	31·78	27·82	36·29	0·162	34·84	23·26	43·77	32·59	<0·001
Time spent cooking – weekend (min)	60·83	44·91	69·04	48·20	<0·001	42·34	37·56	46·15	39·65	<0·001	29·25	37·11	31·15	39·73	0·068	40·17	29·12	48·25	38·74	<0·001
Inclusion of children everyday meal preparation	3·07	1·58	4·07	1·47	<0·001	2·87	1·52	3·31	1·53	<0·001	3·53	1·60	3·53	1·65	0·987	3·37	1·57	3·79	1·57	<0·001
Inclusion of children in baking activities	2·44	1·15	3·38	1·21	<0·001	2·53	1·28	2·97	1·39	<0·001	3·28	1·65	3·38	1·66	0·434	2·87	1·42	3·40	1·44	<0·001

### Differences between low- and high-frequency individuals who included their children in everyday cooking activities during the pandemic

An overview of the statistical differences of low- and high-frequency includers can be seen in Table 3. A brief description of the results follows.

**Table 3:** Differences between high and low includers of children in cooking activities during the pandemic

Variable	IOI					GB					USA							NZ						
	Low	High	*t*/U	95 % CI/*Z*	*P*	Low	High	*t*/U	95 % CI/*Z*	*P*	Low		High		*t*/U	95 % CI/*Z*	*P*	Low		High		*t*/U	95 % CI/*Z*	*P*
	Median	Median				Median	Median				Mean	sd	Mean	sd				Mean	sd	Mean	sd			
Age	40·00	39·00	3132·50	−0·248	0·804	45·00	41·00	9937·50	−2·800	0·005	43·88	17·91	36·20	13·56	2·66	1·97, 13·38	0·009	42·28	12·06	39·29	11·89	1·57	−0·79, 6·77	0·119
CS Conf.	72·00	76·00	2770·50	−1·411	0·158	66·00	72·00	9891·50	−2·857	0·004	64·09	17·23	72·89	19·73	−2·59	−15·54, -2·06	0·011	66·73	17·51	75·60	15·79	−3·38	−14·05, -3·68	0·001
BMI	25·85	24·88	1460·50	−0·531	0·595	25·98	25·61	6192·50	−0·043	0·966	25·66		25·95		430·50	−0·281	0·779	27·73		24·91		1414·50	−0·921	0·357
Fruit during	2·00	2·00	2033·00	−2·681	0·007	2·00	2·00	8900·00	−1·651	0·099	2·00		2·00		820·50	−1·745	0·081	2·00		2·00		2171·50	−2·057	0·040
Vegetable during	2·00	3·00	2070·00	−2·633	0·008	2·00	3·00	8041·50	−3·193	0·001	2·00		3·00		701·50	−3·105	0·002	2·00		3·00		2337·50	−1·626	0·104

	IOI						GB						USA						NZ					
	Low		High				Low		High				Low		High				Low		High			
	*n*	%	*n*	%	*χ* ^2^	*P*	*n*	%	*n*	%	*χ* ^2^	*P*	*n*	%	*n*	%	*χ* ^2^	*P*	*n*	%	*n*	%	*χ* ^2^	*P*
Education																								
Below University	24	38·7	38	61·3			84	58·7	59	41·3			25	59·5	17	40·5			23	37·7	38	62·3		
University	25	21·2	93	78·8	6·300	0·012	73	43·2	96	56·8	7·488	0·006	31	39·7	47	60·3	4·292	0·038	41	39·0	64	61·0	0·029	0·864
Gender																								
Male	6	37·5	10	62·5			77	50·3	76	49·7			20	37·7	33	62·3			34	43·0	45	57·0		
Female	43	26·2	121	73·8		0·380	80	50·6	78	49·4	0·003	0·957	36	54·5	30	45·5	3·334	0·068	30	34·5	57	65·5	1·279	0·258
Age of Learning CS																								
Child or adolescent	20	25·0	60	75·0			69	47·6	76	52·4			25	38·5	40	61·5			23	31·9	49	68·1		
Adult	29	29·0	71	71·0	0·359	0·549	88	52·7	79	47·3	0·810	0·368	31	56·4	24	43·6	3·836	0·050	41	43·6	53	56·4	2·345	0·126
Working from home																								
No	16	43·2	21	56·8			34	61·8	21	38·2			8	42·1	11	57·9			22	37·3	37	62·7		
Yes	24	24·2	75	75·8	4·684	0·030	75	43·4	98	56·6	5·703	0·017	28	39·4	43	60·6	0·044	0·833	29	39·2	45	60·8	0·050	0·823

CS, cooking skills.

#### Sociodemographic variables

There was no significant difference in age between low and high includers in IOI and NZ. However, in GB (*P* = 0·005) and the USA (*P* = 0·010), high includers were significantly younger than low includers. Three regions found a significant relationship between education and inclusion of children in cooking tasks: IOI (*P* = 0·012); GB (*P* = 0·006) and the USA (*P* = 0·038). No relation was seen in NZ. No significant relationship between gender and inclusion of children in cooking was found in any region. Significant relationships between WFH status and inclusion of children in cooking activities were found in IOI (*P* = 0·030) and GB (*P* = 0·017).

#### Cooking-related variables

In three of the regions, high includers had significantly greater CS confidence than low includers: GB (*P* = 0·004), USA (*P* = 0·011) and NZ (*P* = 0·001). This difference was not found in IOI. No significant relations for age of learning CS were found in any region; however, a trend was seen in the USA (*P* = 0·050).

#### Diet quality and health indicators

High includers in both IOI (*P* = 0·007) and NZ (*P* = 0·040) had a significantly greater intake of fruit during the pandemic than the low includers. No significant differences were seen in GB or the USA. Additionally, in three of the regions, high includers had a significantly higher intake of vegetables than the low includers during the pandemic: IOI (*P* = 0·008), GB (*P* = 0·001) and the USA (*P* = 0·002). No difference was seen in NZ. In all four regions, no differences were seen in BMI between low and high includers.

### Significant predictors of including children in cooking during the pandemic

The binomial logistic regression (*χ* ^2^ (11, *n* 516) = 83·12, *P* < 0·001, Cox and Snell R Square 0.149, Negelkerke R Squared 0.201) revealed that country was the strongest predictor of including children in cooking. Those on the IOI were 3·6 times more likely to be high includers compared with the USA (OR = 3·6, 95 % CI 1·7, 7·6). Parent’s level of education was also a strong predictor. Those with a higher level of education were 1·6 times more likely to be high includers (OR = 1·6, 95 % CI 1·1, 2·4). Those with higher CS confidence (OR = 1·02, 95 % CI 1·009, 1·032) and a higher intake of vegetables during the pandemic (OR = 1·3, 95 % CI 1·1, 1·5) were also significantly more likely to be high includers. Females (OR = 0·6, 95 % CI 0·4, 0·9) and older individuals (OR = 0·97, 95 % CI 0·95, 0·99) were significantly less likely to be higher includers. Results not shown (available upon request).

## Discussion

COVID-19 has caused unprecedented societal changes, including a shift to WFH. Anecdotally, there were reports of and encouragement to include children in cooking activities for entertainment and education^(20)^; however, there is no published confirmation of changes in these behaviours. Previously, parents have been reluctant to include their children in kitchen activities due to fear and lack of time^(9,10)^. This research has shown that there were increases in the inclusion of children in both everyday cooking and baking activities in three regions. This change was not seen in the USA sample and may be due to differing movement restrictions implemented in the USA^(14)^, that is, less restrictions and therefore spent less time in the home, thus practices changed less^(15)^. Additionally, a relationship between WFH and including children in cooking was seen in two regions. Undoubtedly, parents have been under increased pressure with caring, work and education responsibilities^(21)^. While they may spend additional time cooking, including children in the process provides an opportunity for learning invaluable life skills that may have positive impacts on their diet quality and track into adulthood^(8,22)^. Furthermore, including children in the cooking process increases willingness to eat vegetables^(23)^ and food in general^(24)^, a key strategy for nutrition and waste reduction in times of increasing food insecurity^(25)^.

This study also investigated predictors for inclusion and differences between those who included their children in cooking more frequently than those who did less frequently during COVID-19. In two regions, age was associated with frequency of including children in cooking and older individuals were less likely to be high includers in the regression. Previously, older adults were shown to have higher CS confidence^(7)^. Here, high includers were shown to have a greater CS confidence. Furthermore, a relationship between education and frequency of including children in cooking activities was found, in line with education being associated with CS confidence^(7)^. Higher parental cooking confidence has additionally been associated with reductions in consumption of ultraprocessed foods by children^(26)^. Therefore, increasing parents’ cooking confidence and reducing their fears of including children in cooking activities may be an area for future focus through parent–child cooking interventions or online cook-along videos during the pandemic, as video technology may help in promoting confidence^(27)^. Additionally, promotion of evidence-based guidance for specific age-appropriate CS may help reduce parental anxiety around their child’s capabilities in the kitchen^(28)^.

Finally, a key finding in this study is that including children in cooking activities was associated with better parental diet quality, greater intakes of fruit in two regions and greater intake of vegetables in three regions than low includers. Additionally, a higher intake of vegetables was a significant predictor of higher inclusion. This could suggest that those with a better diet quality include their children more frequently or including children may increase parents’ diet quality. The latter may be due to parents trying to be positive role models for their children in their preparation and consumption behaviours^(29)^ or it may be the parents trying to choose healthier options or recipes with extra vegetables to expose their children to while including them in cooking.

### Strengths and limitations

Strengths of this research include the cross-continental sampling to gather global perspectives and the use of validated measures where possible to increase comparability and repeatability. The efficient completion of the study in a relatively short timeframe provides a unique ‘snapshot’ of behaviour during a historic global event. However, as with all cross-sectional research, causality cannot be established and some selection bias may have occurred, for example, a greater number of IOI sample were recruited using social media which may have contributed towards the greater number of females. The use of a convenience sample may affect generalisability of the results; however, the use of multiple countries and a range of participants (wide age ranges, both male and female – cooking research tends to focus on the mother) may help to reduce this affect. Future research using a greater number of participants across each country would allow for multivariate analyses for each country.

## Conclusions

This study has confirmed previous anecdotal evidence of increases in including children in cooking activities during the COVID-19 pandemic. CS confidence was associated with a higher frequency of including children in cooking activities. Additionally, while there are several benefits associated with the inclusion of children in cooking, such as providing life skills and increases in diet quality, a higher intake of vegetables by parents who included children more frequently in cooking activities was seen. With continued lockdowns due to COVID-19 and perhaps future increased flexibility in WFH, including children in cooking activities should be a key public health message for both children and parents.

## Supplementary material

For supplementary material accompanying this paper visit http://dx.doi.org/10.1017/S1368980021001932. click here to view supplementary material
